# Microstructural Evolution and Mechanical Properties of Non-Equiatomic (CoNi)_74.66_Cr_17_Fe_8_C_0.34_ High-Entropy Alloy

**DOI:** 10.3390/ma15041312

**Published:** 2022-02-10

**Authors:** You Sub Kim, Hobyung Chae, E-Wen Huang, Jayant Jain, Stefanus Harjo, Takuro Kawasaki, Sun Ig Hong, Soo Yeol Lee

**Affiliations:** 1 Department of Materials Science and Engineering, Chungnam National University, Daejeon 34134, Korea; usube2012@cnu.ac.kr (Y.S.K.); highteen5@cnu.ac.kr (H.C.); 2 Department of Materials Science and Engineering, National Yang Ming Chiao Tung University, Hsinchu 30013, Taiwan; ewenhuang@nctu.edu.tw; 3 Department of Materials Science and Engineering, Indian Institute of Technology Delhi, New Delhi 110016, India; jainjayan@gmail.com; 4 J-PARC Center, Japan Atomic Energy Agency, Ibaraki 319-1195, Japan; stefanus.harjo@j-parc.jp (S.H.); takuro.kawasaki@j-parc.jp (T.K.)

**Keywords:** high-entropy alloy, mechanical property, stacking fault energy, neutron diffraction

## Abstract

In this study, we manufactured a non-equiatomic (CoNi)_74.66_Cr_17_Fe_8_C_0.34_ high-entropy alloy (HEA) consisting of a single-phase face-centered-cubic structure. We applied in situ neutron diffraction coupled with electron backscattered diffraction (EBSD) and transmission electron microscopy (TEM) to investigate its tensile properties, microstructural evolution, lattice strains and texture development, and the stacking fault energy. The non-equiatomic (CoNi)_74.66_Cr_17_Fe_8_C_0.34_ HEA revealed a good combination of strength and ductility in mechanical properties compared to the equiatomic CoNiCrFe HEA, due to both stable solid solution and precipitation-strengthened effects. The non-equiatomic stoichiometry resulted in not only a lower electronegativity mismatch, indicating a more stable state of solid solution, but also a higher stacking fault energy (SFE, ~50 mJ/m^2^) due to the higher amount of Ni and the lower amount of Cr. This higher SFE led to a more active motion of dislocations relative to mechanical twinning, resulting in severe lattice distortion near the grain boundaries and dislocation entanglement near the twin boundaries. The abrupt increase in the strain hardening rate (SHR) at the 1~3% strain during tensile deformation might be attributed to the unusual stress triaxiality in the {200} grain family. The current findings provide new perspectives for designing non-equiatomic HEAs.

## 1. Introduction

Multi-component alloys, called high-entropy alloys (HEAs) are composed of at least four elements and form a solid solution with a single-phase crystal structure [1,2,3,4,5]. Much attention has been given to HEAs for overcoming a strength-ductility trade-off in their mechanical properties because of their exceptional strength, ductility and fracture toughness. In addition, HEAs play an important role as the second phase in the manufacturing of new alloys, since they improve strength-ductility synergy [6]. These properties come from their lattice structure distortion, sluggish diffusion kinetics, the cocktail effect and the evolution of deformation twinning [3,7,8,9,10,11,12]. One widely studied research project on CoNiCrFe HEAs reported that a single-phase CoNiCrFe possesses high tensile strength and ductility [13]. Furthermore, mechanical properties are significantly improved at cryogenic temperatures due to the evolution of nano-twins [14,15,16]. Zhou et al. [17] reported that C-containing (1.21 at%) CoNiCrFe enhanced the yield strength and tensile strength by 9% and 7%, respectively, compared to CoNiCrFe. Many HEA researchers have demonstrated that the formation of HEAs is not highly dependent on the maximum configurational entropy via equiatomic ratios of elements. In addition, they reported that entropy is not the most dominant factor in the strength of solid solution-strengthened HEAs [15,18,19]. These arguments led to more active research on non-equiatomic HEAs, compared to equiatomic HEAs [20].

It is well known that the deformation mechanisms of face-centered-cubic (FCC) HEAs depend on stacking fault energy (SFE), which is a useful parameter to classify the deformation mechanisms for slip (>45 mJ/m^2^) to twinning (20–45 mJ/m^2^) and phase transformation (<20 mJ/m^2^) [21,22,23,24,25,26,27,28]. Liu et al. [29] reported that the SFE of CoNiCrFe is ~27 mJ/m^2^ at room temperature, as calculated by TEM. Wang et al. [14] reported that the SFE of CoNiCrFe decreased from 32.5 mJ/m^2^ (at 293 K) to 13 mJ/m^2^ (at 77 K). Generally, SFE is calculated by TEM, ab initio calculation and diffraction-based techniques (X-ray or neutron). However, ex situ TEM calculates the SFE in a very localized area and ab initio calculation contains many factors and assumptions that significantly influence the variation of results. X-ray diffraction can provide the SFE based on the statistical information measured near the surface of metallic alloys; however, due to its low penetration depth in structural alloys, X-ray diffraction is limited in representing bulk property. On the other hand, neutron diffraction has high penetration depth (a few cm) in metallic alloys. Thus, it is well suited to providing the volume-averaged statistical SFE in thick metallic alloys.

In this study, we manufactured a new design of non-equiatomic precipitated (CoNi)_74.66_Cr_17_Fe_8_C_0.34_ HEAs for mechanical property improvement over equiatomic CoNiCrFe and other types of FCC single-phase non-equiatomic HEAs. In situ neutron diffraction was conducted to examine tensile behavior and its related deformation mechanisms, by performing the peak profile analysis to calculate the SFE of this alloy system. Our work can help researchers design a new alloy system in terms of non-equiatomic HEAs and overcome the limitations of conventional HEA design.

## 2. Materials and Methods

### 2.1. Specimen Preparations

**Figure 1:**
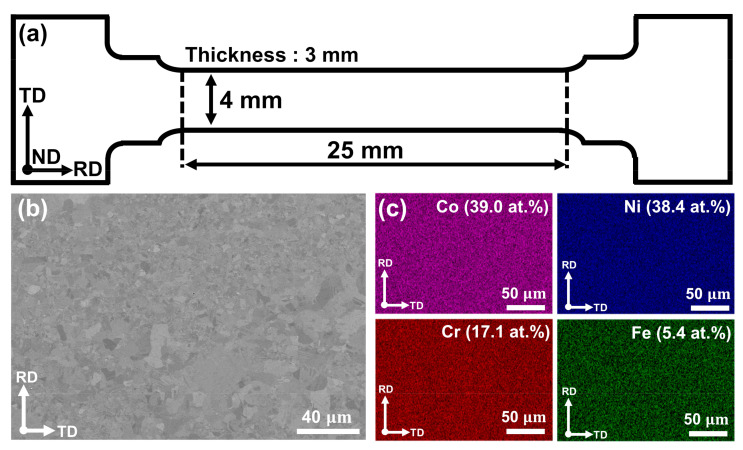
(**a**) The geometry of tensile specimen, (**b**) the microstructure and (**c**) EDS analysis using scanning electron microscopy.

Figure 1 shows the geometry of the tensile specimen and the microstructure, and chemical composition measured from scanning electron microscopy (SEM, Merlin, ZEISS, Oberkochen, Germany) and energy dispersive X-ray spectroscopy (EDS, X-Max^N^, Oxford, UK) for (CoNi)_74.66_Cr_17_Fe_8_C_0.34_ HEA. The detailed chemical composition of the alloy was analyzed by the inductively coupled plasma (ICP) method, whose information is provided in Table 1. Initial ingots with a size of 40 × 110 × 140 mm^3^ were manufactured by vacuum induction melting. The ingots were cut to the size of 30 × 40 × 15 mm^3^ and homogenized in a vacuum at 1200 °C for 6 h. After homogenization, the ingots were rolled into a plate with a thickness of 3 mm at room temperature (RT). The final plate was annealed at 850 °C for 1 h to obtain the recrystallized microstructure.

### 2.2. Microstructure Characterization

The microstructure of the rolled plate was investigated on the plane parallel to the rolling direction. The specimens were mechanically polished under 6, 3 and 1 μm by using a diamond suspension, and the mirror-finished surface was electrically polished for electron back-scattered diffraction (EBSD, NordlysNano, Oxford, UK) analysis. The conditions of electropolishing were as follows: a voltage of 5 V, an exposure time of 20 s at RT and a nitric acid (35%) solution. Transmission electron microscope (TEM, JEM-2100F, JEOL, Tokyo, Japan) specimens were prepared by a twin-jet electropolishing method using a solution of 90% methanol and 10% perchloric acid with a 25 V at RT.

### 2.3. In Situ Neutron Diffraction Experiments

**Figure 2:**
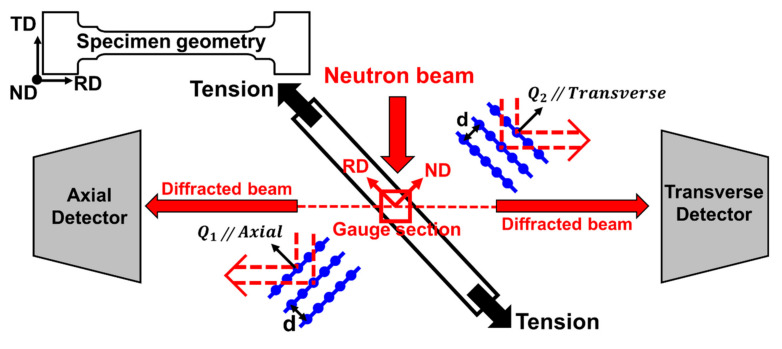
Schematic of time-of-flight (TOF) in situ neutron diffraction geometry at the spallation neutron source.

In situ neutron diffraction experiments were conducted under monotonic tensile loading with a strain rate of $2\times 10^{-5}\;s^{-1}$ using the TAKUMI diffractometer in the Materials and Life Science Experimental Facility (MLF) of the Japan Proton Accelerator Research Complex (J-PARC, Tokai-mura, Ibaraki, Japan) [30]. Figure 2 is a schematic of time-of-flight (TOF) in situ neutron diffraction geometry at the spallation neutron source. The specimen was positioned at 45° and two detectors (axial and transverse) were located at ±90° from the incident neutron beam. Since the neutron diffraction data was collected by the two detector banks, the entire diffraction patterns, whose scattering vectors were parallel (axial detector) and perpendicular (transverse detector) to the tensile loading direction (shown in Figure 2), were acquired under in situ tensile loading. From this, we received information on lattice strains for various crystallographic orientations. We gathered information on texture from relative intensity variations, and on the microstructural changes during plastic deformation from diffraction peak profile analysis. 

### 2.4. Peak Profile Analysis

Stacking fault energy (SFE, mJ/m^2^) is defined as units of energy per area, which is the parameter of how easily perfect dislocation dissociates into two Shockley partial dislocations 1/6 < 112 > on the FCC (111) plane. The passage of Shockley partial dislocation on the successive (111) planes generates multi-layer intrinsic stacking faults, which can formulate the initiation sites of twins. The stacking faults, expressed by diffraction peak shift (∆TOF/∆d) and diffraction peak broadening, induce a defect scattering in neutron diffraction and the evolution of inhomogeneous strain (lattice distortion) in the matrix during tensile loading. The relationship between the diffraction peak shift and stacking fault probability (SFP, $P_{sf}$) is well described by Warren [31] (Equation (1)). In addition, Warren and Averbach [32] formulated the broadened profile into the Fourier transformation coefficients. Balzar et al. [33,34] reported a size-strain broadening equation using a simple Voigt function. The Double-Voigt method can characterize the strain (distortion) broadening ($\beta_{D}$) depending on the diffraction angle, while the size (domain) broadening ($\beta_{S}$) cannot. The mean square strain (MSS, $\langle \xi_{50}^{2}\rangle$), which is caused by inhomogeneous strain quantity, is calculated using Equation (2). Reed and Scharmms proposed the relationship between SFE, SFP and MSS [35] (Equation (3)), which can be calculated by neutron diffraction peak profile analysis. In this work, the Rietveld method and single peak fitting for peak profile analysis are used to examine the microstructural evolution, such as dislocation, stacking faults, twinning and microstrain [34]. Several terms used in Equations (1)–(3) are defined as follows: $d_{hkl}^{0}$ is the initial interplanar spacing and $d_{hkl}$ is the interplanar spacing of *hkl* planes under loading. $\beta_{LD}^{\;*}$ and $\beta_{GD}^{\;*}$ are integral breaths of Lorentzian and Gaussian distortion, converting from real space to reciprocal space, respectively. s (1/d=2sinθ/λ, ${\mathrm{nm}}^{-1}$) is the parameter in reciprocal space. $a_{0}$ is the lattice parameter, and $C_{11}$, $C_{12}$ and $C_{44}$ are the elastic stiffness coefficients. $a_{0}$ is 0.3545 nm, $C_{11}$ is 271 GPa, $C_{12}$ is 175 GPa and $C_{44}$ is 189 GPa [14].

(1) $$\mathrm{SFP}\left(P_{sf}\right)=\frac{32\pi }{3\sqrt{3}}\left[{\left(\frac{d-d_{0}}{d_{0}}\right)}_{222}-{\left(\frac{d-d_{0}}{d_{0}}\right)}_{111}\right]$$

(2) $$\mathrm{MSS}\langle \xi_{\;50}^{2}\rangle =\left(\beta_{LD}^{\;*}/s_{0}^{2}\right)/50\pi^{2}+\left(\beta_{GD}^{\;2*}/s_{0}^{2}\right)/2\pi$$

(3) $$\mathrm{SFE}=\frac{6.6a_{0}}{\pi \sqrt{3}}{\left(\frac{2C_{44}}{C_{11}-C_{12}}\right)}^{-0.37}\frac{\langle \xi_{\;50}^{2}\rangle {}_{111}}{P_{sf}}\left(\frac{C_{44}+C_{11}-C_{12}}{3}\right)$$

## 3. Results

### 3.1. Stress–Strain Response in Tension

**Figure 3:**
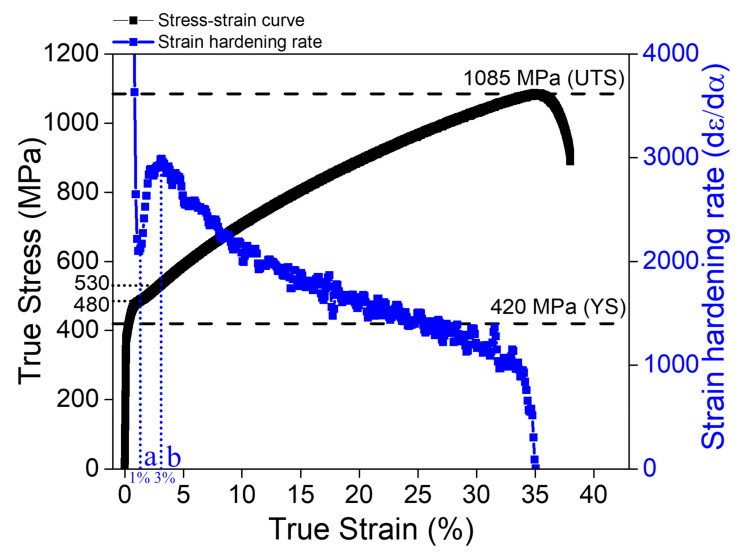
Stress–strain response and strain hardening rate of the non-equiatomic (CoNi)_74.66_Cr_17_Fe_8_C_0.34_ HEA.

Figure 3 shows the stress–strain response of the (CoNi)_74.66_Cr_17_Fe_8_C_0.34_ HEA. The non-equiatomic (CoNi)_74.66_Cr_17_Fe_8_C_0.34_ exhibited a relatively higher ultimate tensile strength (UTS) and moderate ductility compared to the equiatomic CoNiCrFe and single-phase non-equiatomic FCC HEAs [36]. The UTS and ductility of (CoNi)_74.66_Cr_17_Fe_8_C_0.34_ were 771 MPa and 46%, respectively, in the engineering stress–strain curve. The strain hardening rate (SHR) of (CoNi)_74.66_Cr_17_Fe_8_C_0.34_ (the blue square in Figure 3) showed unusual behavior at the very early stage of deformation, such that the SHR suddenly dropped down to the strain of 1% (corresponding to 480 MPa) and then abruptly increased up to the strain of 3% (corresponding to 530 MPa), after which the SHR gradually decreased until fracture.

### 3.2. Lattice Strains and Intensity Variations

**Figure 4:**
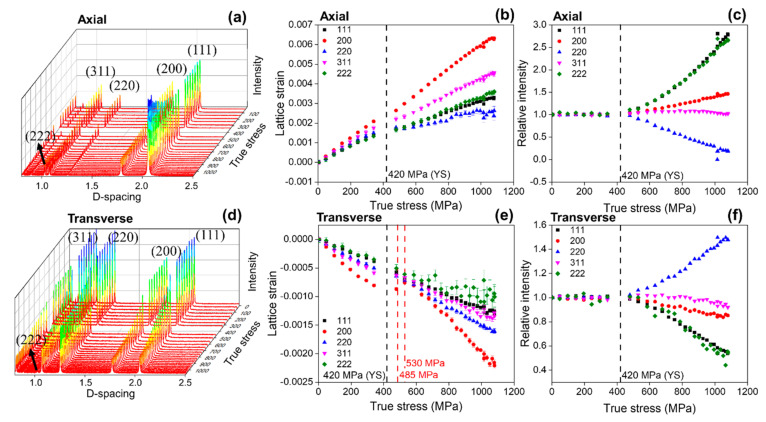
In situ neutron diffraction experiment results: the evolution of (**a**) sequential diffraction patterns, (**b**) lattice strains, (**c**) diffraction peak intensities during tensile loading in the axial direction, (**d**) sequential diffraction patterns, (**e**) lattice strains and (**f**) diffraction peak intensities during tensile loading in the transverse direction.

Figure 4 shows the in situ evolution of (a) sequential neutron diffraction patterns, (b) lattice strains and (c) diffraction peak intensities during tensile loading for the non-equiatomic (CoNi)_74.66_Cr_17_Fe_8_C_0.34_ HEA. The studied alloy exhibited a face-centered-cubic (FCC) crystal structure without any phase transformation during tensile deformation, as revealed in both axial and transverse diffraction patterns (Figure 4a,d). The different slope of {hkl} lattice strains with an increase in applied stress indicates a distinct elastic and plastic anisotropy of the alloy in the lattice level. The soft grain families ({220}, {111}), where the slope of lattice strains decreased under applied stress, showed micro-yielding before the macroscopic yield strength (YS), which indicates that those soft grains began to deform plastically at the stress level. On the other hand, the {200} grain family manifested the hardest grain orientation, indicating that {200} grain is the most difficult to deform plastically among the grains examined. The order of {200}, {311}, {111} and {220} in harder grain orientations was typical in FCC entropy alloys, and the new alloy system also followed a similar trend; however, the {200} grain behavior in the transverse direction (Figure 4e) revealed a sudden increase in lattice strains from 478 MPa to 530 MPa, corresponding to a sharp increase of strain hardening rate, as shown in Figure 3. This unusual phenomenon will be further examined in the Discussion section. The diffraction intensities of all the grain families did not change before the YS, after which the intensities of {111} and {222} grain orientations continuously increased to almost three times larger than the initial intensities. This indicates that during plastic deformation, the grains rotated toward the {111} orientation along the axial tensile loading direction (Figure 4c). In contrast, the evolution of the diffraction intensities in the transverse direction (Figure 4f) was completely the opposite of those in the axial direction (Figure 4c).

### 3.3. Microstructural Evolution

**Figure 5:**
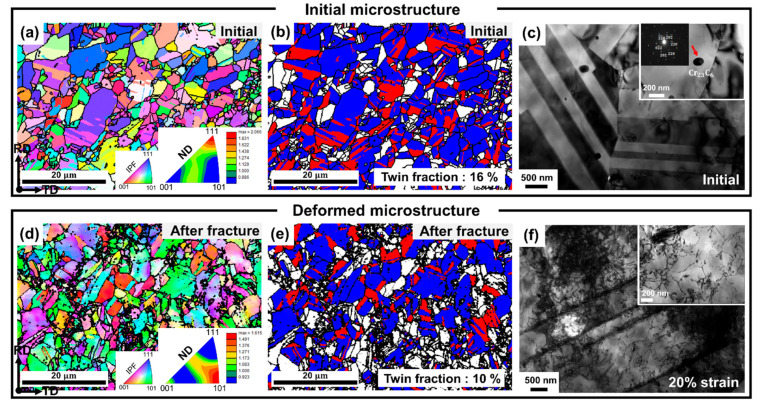
The microstructures measured from EBSD and TEM: (**a**–**c**) are the initial microstructure, (**d**–**e**) are the microstructure after fracture and (**f**) is the microstructure at 20% strain.

Figure 5 shows the microstructures of the (CoNi)_74.66_Cr_17_Fe_8_C_0.34_ HEA before and after plastic deformation. In the initial state, the alloy possessed various grain sizes (10% below 1 μm, 71% for 1~5 μm and 19% above 5 μm) and annealing twins due to recrystallization (Figure 5a,b). The initial microstructure exhibited chromium carbide (Cr_23_C_6_) with a size of 100~150 nm, and the carbides were located near the grain and twin boundaries (Figure 5c). The alloy manifested {111} preferred orientation in ND (Figure 5a) in the initial state, but the texture changed from {111} to {101} orientation in the plastically deformed sample (Figure 5d), which aligned well with the increase of the {220} diffraction peak in the transverse direction (Figure 4f). In contrast with the mechanical twinning to the plastic deformation, dislocations played a significant role in the plastic deformation, resulting in severe lattice distortion near the grain boundaries (Figure 5d,e), and causing dislocations to become entangled near the twin boundaries (Figure 5f).

### 3.4. Stacking Fault Energy

**Figure 6:**
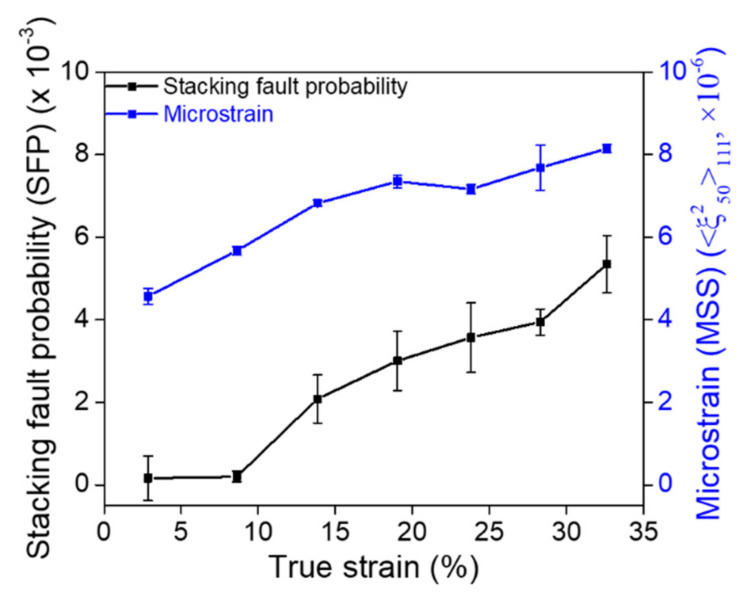
Development of stacking fault probability (black color) and microstrain (blue color) as a function of true strain during the tensile test.

The development of stacking fault probability (SFP) and microstrain as a function of true strain is presented in Figure 6. These results were obtained from peak profile analysis based on the data measured by an in situ neutron diffraction experiment [37]. The stacking faults evolved during tensile deformation induce the difference in lattice strains between the {111} and {222} orientations, thereby resulting in a gradual increase in the SFP. In addition, distortion (inhomogeneous strain) that was severely induced in the lattice during plastic deformation, increased the mean square strain (MSS). With a combination of SFP and MSS, the SFE was calculated using Equation (3). The average SFE of the (CoNi)_74.66_Cr_17_Fe_8_C_0.34_ HEA was 49.7 mJ/m^2^ at about 20% strain. This indicates that the deformation mechanism of the alloy was mainly controlled by a dislocation glide, as supported by the TEM results (Figure 5e,f) and other investigations on austenitic alloys [21,22,23,24,25,26,27,28].

## 4. Discussion

Atomic size mismatch (δ) and electronegativity mismatch (∆χ) are classical factors that determine the stability of a solid solution and the intrinsic properties of HEAs [38]. δ is defined by $\delta =\sqrt{\sum_{i=1}^{n}C_{i}{\left(1-\frac{r_{i}}{\bar{r}}\right)}^{2}}$, where $C_{i}$ is the elemental fraction of each component, $r_{i}$ is the atomic radius of each element, and $\bar{r}$ is the mean radius of the alloy [39]. A theoretical δ of the non-equiatomic (CoNi)_74.66_Cr_17_Fe_8_C_0.34_ HEA was 1.17%, and for the equiatomic CoNiCrFe HEA it was 1.18%. Both alloys exhibited a similar δ value. Thus, it was difficult to account for the enhanced mechanical properties of (CoNi)_74.66_Cr_17_Fe_8_C_0.34_ with the concept of a solid solution and lattice distortion strengthening effect. ∆χ is defined by $∆\chi =\sqrt{\sum_{i=1}^{n}C_{i}{\left(\chi_{i}-\bar{\chi }\right)}^{2}}$, where $C_{i}$ is the elemental fraction of each component, $\chi_{i}$ is the electronegativity of each element, and $\bar{\chi }$ is the mean of electronegativity for the alloy [40]. The ∆χ of the non-equiatomic (CoNi)_74.66_Cr_17_Fe_8_C_0.34_ HEA and the equiatomic CoNiCrFe HEA were 8.90% and 9.67%, respectively. A lower ∆χ indicates a stable state of electrons that can maintain more stable solid solutioning. Consequently, the non-equiatomic (CoNi)_74.66_Cr_17_Fe_8_C_0.34_ HEA manifests a more stable state of solid solution than the equiatomic CoNiCrFe HEA, which might relate to the improvement in the mechanical properties of the (CoNi)_74.66_Cr_17_Fe_8_C_0.34_ HEA. Another possible reason for the enhanced strength of the non-equiatomic (CoNi)_74.66_Cr_17_Fe_8_C_0.34_ HEA is attributed to the precipitation-strengthened effect caused by the formation of Cr_23_C_6_ (Figure 5c), as well as grain refinement [41]. This is because the carbides act as effective barriers for dislocation movement. In this regard, the (CoNi)_74.66_Cr_17_Fe_8_C_0.34_ HEA revealed a higher yield and tensile strength than the equiatomic CoNiCrFe HEA.

The average SFE of the (CoNi)_74.66_Cr_17_Fe_8_C_0.34_ HEA (~50 mJ/m^2^) was higher than the equiatomic CoNiCrFe HEA (~30 mJ/m^2^) [14,29], which was due to the different composition of alloys from major elements. The SFE of Ni (79~90 mJ/m^2^) is higher than other elements. Cr, Co and Fe play a vital role in decreasing the SFE of Ni. Cr is especially more effective than Fe [42]. Therefore, the SFE of the (CoNi)_74.66_Cr_17_Fe_8_C_0.34_ HEA was higher than that of the equiatomic CoNiCrFe HEA, due to a higher amount of Ni and a lower amount of Cr. The higher SFE of the (CoNi)_74.66_Cr_17_Fe_8_C_0.34_ HEA mainly led to more active dislocation motion than mechanical twinning, as confirmed by EBSD and TEM (Figure 5d–f).

The non-equiatomic (CoNi)_74.66_Cr_17_Fe_8_C_0.34_ HEA exhibited three different stages of SHR behavior during tensile deformation. The SHR sharply dropped down to 1% strain and increased rapidly up to 3% strain, after which it gradually decreased until fracture (Figure 3). A similar unusual SHR behavior was reported in an Fe-20%Mn-1.2%C TWIP steel [43], and it was demonstrated that the S-shape curve is related to the nucleation and growth mechanisms of twins during plastic deformation. When considering that the initial microstructure of the studied alloy already contained the ~16% twin fraction and that there were no significant changes in the twinning volume fraction after the failure (Figure 5e), the contribution of twinning activities would be negligible in rationalizing the rapid increase of the SHR in the 1~3% strain. Rather, the drastic increase of the SHR at the 1~3% strain might be correlated with the lattice strain behavior of the {200} grain family in the transverse direction (Figure 4e). Based on the axial lattice strain changes of the {200} orientation, where the grain became harder in the plastic regime, the corresponding transverse {200} lattice strain should move toward the more negative slope, as observed in a more rapid drop in the transverse strains above the stress value of 530 MPa (Figure 4e). However, the transverse lattice strain of the {200} orientation even increased to the lower value, which means that relatively high tensile stresses that can overcome the reduction of the lattice strains by the Poisson’s ratio effect, should be imposed in the transverse direction (perpendicular to the tensile loading) in the strain range of 1~3%. It is thought that the unusual stress triaxiality shown in the {200} grain family could be a consequence of the interactions between dislocations and twins and/or carbides; however, further investigations are necessary to determine the exact cause.

## 5. Conclusions

We manufactured a non-equiatomic (CoNi)_74.66_Cr_17_Fe_8_C_0.34_ high-entropy alloy with a single-phase FCC structure. We used in situ neutron diffraction coupled with EBSD and TEM to investigate tensile properties, microstructural evolution, lattice strains, texture development and stacking fault energy. Our main conclusions are drawn as follows:

1. The initial microstructure of the non-equiatomic (CoNi)_74.66_Cr_17_Fe_8_C_0.34_ possessed various grain sizes, annealing twins, {111} texture and nano-size chromium carbide. Compared to the equiatomic CoNiCrFe HEA, the non-equiatomic (CoNi)_74.66_Cr_17_Fe_8_C_0.34_ HEA manifested a lower electronegativity mismatch, indicating a more stable state of solid solution, which improves its mechanical properties.
2. Compared to the equiatomic CoNiCrFe HEA and other FCC-based non-equiatomic HEAs, the non-equiatomic (CoNi)_74.66_Cr_17_Fe_8_C_0.34_ HEA revealed a relatively higher tensile strength (770 MPa) and moderate ductility (46%), due to both a stable solid solution and precipitation-strengthened effects.
3. The sudden increase in the SHR at the 1~3% strain during tensile deformation might be attributed to the unusual stress triaxiality in the {200} grain family, as revealed from the sharp increase in the transverse lattice strains.
4. The average stacking fault energy of the (CoNi)_74.66_Cr_17_Fe_8_C_0.34_ HEA determined from neutron diffraction peak profile analysis was 49.7 mJ/m^2^. The higher SFE of the (CoNi)_74.66_Cr_17_Fe_8_C_0.34_ HEA led to the active motion of dislocations relative to mechanical twinning, resulting in severe lattice distortion near the grain boundaries and dislocation entanglement near the twin boundaries.

## Figures and Tables

**Table 1:** Chemical composition of the (CoNi)_74.66_Cr_17_Fe_8_C_0.34_ alloy.

Element (at.%)	Co	Ni	Cr	Fe	C
(CoNi)_74.66_Cr_17_Fe_8_C_0.34_	36.27	38.39	17	8	0.34

## Data Availability

The data required to reproduce these findings cannot be shared at this time as the data also forms part of an ongoing study.
